# Integrated systems analysis of salivary gland transcriptomics reveals key molecular networks in Sjögren’s syndrome

**DOI:** 10.1186/s13075-019-2082-9

**Published:** 2019-12-19

**Authors:** Hong Ki Min, Su-Jin Moon, Kyung-Su Park, Ki-Jo Kim

**Affiliations:** 1 Division of Rheumatology, Department of Internal Medicine, Konkuk University Medical Center, Seoul, Republic of Korea; 2 Division of Rheumatology, Department of Internal Medicine, Uijeongbu St. Mary’s Hospital, College of Medicine, The Catholic University of Korea, Seoul, Republic of Korea; 3 Division of Rheumatology, Department of Internal Medicine, St. Vincent’s Hospital, College of Medicine, The Catholic University of Korea, Seoul, Republic of Korea

**Keywords:** Sjögren’s syndrome, Salivary gland, Gene expression profiles, Unsupervised clustering, Key driver analysis

## Abstract

**Background:**

Treatment of patients with Sjögren’s syndrome (SjS) is a clinical challenge with high unmet needs. Gene expression profiling and integrative network-based approaches to complex disease can offer an insight on molecular characteristics in the context of clinical setting.

**Methods:**

An integrated dataset was created from salivary gland samples of 30 SjS patients. Pathway-driven enrichment profiles made by gene set enrichment analysis were categorized using hierarchical clustering. Differentially expressed genes (DEGs) were subjected to functional network analysis, where the elements of the core subnetwork were used for key driver analysis.

**Results:**

We identified 310 upregulated DEGs, including nine known genetic risk factors and two potential biomarkers. The core subnetwork was enriched with the processes associated with B cell hyperactivity. Pathway-based subgrouping revealed two clusters with distinct molecular signatures for the relevant pathways and cell subsets. Cluster 2, with low-grade inflammation, showed a better response to rituximab therapy than cluster 1, with high-grade inflammation. Fourteen key driver genes appeared to be essential signaling mediators downstream of the B cell receptor (BCR) signaling pathway and to have a positive relationship with histopathology scores.

**Conclusion:**

Integrative network-based approaches provide deep insights into the modules and pathways causally related to SjS and allow identification of key targets for disease. Intervention adjusted to the molecular traits of the disease would allow the achievement of better outcomes, and the BCR signaling pathway and its leading players are promising therapeutic targets.

## Background

Sjögren’s syndrome (SjS) is a systemic autoimmune disease with a specific predisposition for causing inflammation of the exocrine glands, predominantly the salivary and lacrimal glands [1, 2]. This exocrinopathy usually results in dryness of the mouth and eyes, fatigue, and joint pain, and has a negative effect on quality of life [1, 2]. Despite decades of intensive research, current management is limited to the treatment of sicca symptoms and no effective drug has yet been shown to modify the underlying etiopathogenesis [1, 2]. This is ascribed partly to the wide spectrum of glandular and extraglandular symptoms, the heterogeneity of clinical trial participants, and a lack of reasonable outcome measures to evaluate the treatment response in patients with SjS [1, 3]. In addition, there are substantial gaps in our knowledge regarding the mechanistic basis of SjS progression and molecular stratification applicable to clinical practice.

The pathological hallmarks of SjS are the extensive infiltration of mononuclear cells into salivary glands and the activation of salivary gland epithelial cells (SGECs) [1, 4, 5]. Activation of toll-like receptor (TLR) signaling in the gland epithelium causes the production of autoantigens, the upregulation of immune-competent molecules, apoptosis, and epithelial dysfunction. Autoantigens can be released from SGECs and presented to immune cells. CD4^+^ T cells differentiate into follicular helper T(T_FH_) cells, which are involved in the ectopic formation of germinal centers in salivary glands and contribute to the survival and autoantibody production of B cells. Interaction between SGECs and B cells promotes B cell differentiation. Chemokines and cytokines such as interferon (IFN)-γ, interleukin (IL)-6, IL-12, IL-17, and BAFF (B cell-activating factor, also known as TNFLSF13B or BLyS) have key regulatory roles in these responses. The initiation and progression of SjS is not the sum of fragmentary states but a chain reaction mediated by multiple coordinated molecular pathways and cellular activities. In the light of this complexity, new approaches are needed to boost understanding of the molecular evolution and cellular networks of clinical trajectories of SjS.

Systems biology approaches provide powerful means to elucidate the coordinated molecular processes underlying the pathophysiology of complex diseases [6–9], and we have recently reported novel molecular clustering and pathological characterization for treatment response for rheumatoid arthritis and systemic sclerosis using systems biology and machine learning methods [10, 11]. Here, we sought to integrate salivary gland transcriptomic data in the context of active SjS to construct a model of the pathological inflammatory component of SjS. We systemically searched the salivary gland transcriptomics datasets in the biomedical literature and public data repositories and integrated them, which increased sample size and allowed for the identification and validation of robust and reproducible signatures of the SjS phenotype. We used this dataset to separate expression-driven subgroups and understand the key cellular and molecular elements in each group. Next, we compared our findings from SjS patients with an SjS mouse model and investigated the clinical relevance of the subgroups in terms of treatment response. Finally, we applied an integrative network-based approach and a Bayesian inference to identify the key causal regulators of the disease module.

## Methods

### Systematic search and data collection

**Fig. 1:**
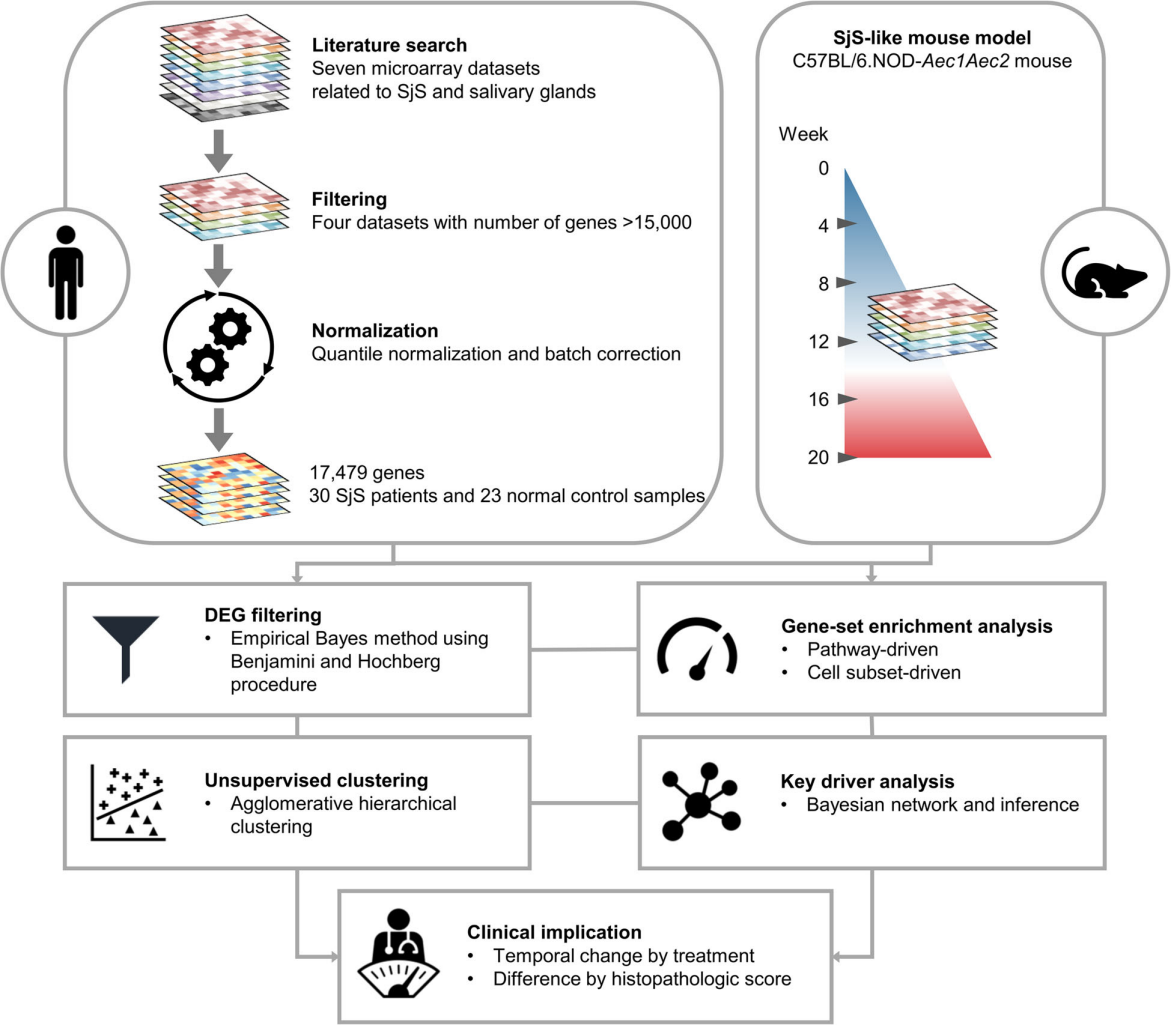
Overview of data processing steps. A total of seven datasets were retrieved from the public data registries (GEO and ArrayExpress). Four datasets were selected for integrated analysis, including samples from 30 patients with Sjögren’s syndrome (SjS) and 23 normal controls, covering 17,479 genes. The merged dataset was normalized using quantile normalization, and its batch effect was further corrected. Filtration of differentially expressed genes (DEGs), gene-set enrichment analysis (GSEA), unsupervised clustering, and key driver analysis (KDA) were performed according to the established methodology, and the clinical and molecular implications of the results were explored

We used the keywords “Sjögren’s syndrome,” “salivary gland,” “transcriptomics or microarray,” and “dataset” in PubMed, Google Scholar, and public data repositories (GEO, ArrayExpress) to find relevant publications to the topic of salivary gland gene signatures of patients with SjS (Fig. 1). We retrieved all publications that were accompanied by high-throughput datasets (seven datasets in total). To secure the largest size of genes and samples, the datasets measuring over 15,000 genes were selected, finally resulting in four datasets (GSE7307, GSE23117, GSE40611, GSE80805). The aggregated number of SjS patients and normal healthy control (NC) was 30 and 23, respectively, and all SjS patients fulfilled the endorsed classification criteria for SjS [12, 13].

### Data normalization and removal of batch effects

All datasets were profiled for gene expression using the Affymetrix array and the Robust Multi-array Average method was applied on the image data for a set of replicates for background correction, normalization, and probe-set summarization. Residual technical batch effects arising due to heterogeneous data integration were corrected using the ComBat function [14, 15]. Quality assurance and distribution bias was evaluated by principal component analysis. After preprocessing, the gene expression profiles have a significant reduction of systematic, dataset-specific bias in comparison with the same dataset before normalization and batch correction (Additional file 1: Figure S1).

### Filtering of differentially expressed genes

In order to identify the differentially expressed genes (DEGs), we used limma R package, a software designed for the analysis of gene expression involving comparisons between many gene targets simultaneously [16]. limma borrows information across genes by fitting linear models to overcome the problem of small sample size and complex experimental design. Briefly, (1) linear models were fitted for expression data of each transcript, (2) empirical Bayes method was used to borrow information across genes, (3) *P* values were adjusted by the Benjamin Hochberg method, and (4) the adjusted *P* value cutoff of 0.01 was then applied.

### Construction of protein-protein interaction network

To assess the interconnectivity of DEGs in the SjS salivary gland samples, we constructed a protein-protein network based on the human interactome database [17]. In the network, nodes and edges represent genes and functional or physical relationships between them, respectively. Graph theory concepts such as degree, closeness, and betweenness were employed to assess the topology of this network. Hub molecules were defined as the shared genes in top 10% with the highest rank in each arm of the three centrality parameters [18].

### Functional and gene set enrichment analysis

We performed functional enrichment analysis focusing on the list of upregulated DEGs using the Enrichr software [19]. Gene ontology (GO)–biological process terms were regarded significant if the adjusted *P* value is lower than 0.01. GO terms irrelevant to salivary gland were filtered out. Gene set enrichment analysis (GSEA) analysis was carried out using the GSEA software from the Broad Institute to assess the overrepresentation of SjS-related gene sets [20]. The enrichment results were visualized with the Enrichment Map format, where nodes represent gene sets and weighted links between the nodes represent an overlap score depending on the number of genes two gene sets share (Jaccard similarity coefficient) [21]. To intuitively identify redundancies between gene sets, the nodes were connected if their contents overlap by more than 25%.

To test for gene enrichment in individual samples, we used a single sample version of gene set enrichment analysis (ssGSEA), which defines an enrichment score as the degree of absolute enrichment of a gene set in each sample within a given data set [22]. The gene expression values for a given sample were rank-normalized, and an enrichment score was produced using the Empirical Cumulative Distribution Functions of the genes in the signature and the remaining genes. This procedure is similar to the GSEA technique, but the list is ranked by absolute expression in one sample.

### Inference of cell types in gene expression profiles

In order to deconvolute the cellular composition of the two clusters in our data, we used an algorithm called xCell [23], a powerful machine learning framework trained on the profiles of 64 immune and stroma cell datasets, for generating cell type enrichment scores and adjusting them to cell type proportions.

### Unsupervised hierarchical clustering and determination of the optimal number of clusters

To classify the SjS patients into subgroups based on their molecular signatures, we used the agglomerative hierarchical clustering method, a commonly used unsupervised learning tool [24]. An agglomerative approach begins with each observation in a distinct cluster. Then, the similarity (or distance) between each of the clusters is computed and the two most similar clusters are merged into one. It successively repeats to merge clusters together and update the proximity matrix until only a single cluster remains. Agglomerative hierarchical clustering was performed with the dissimilarity matrix given by Euclidean distance and the average linkage score was used to join similar clusters [24]. The Euclidean distance is the ordinary straight-line distance between two points in Euclidean space, and the larger the distance between two clusters, the more distinct it is. The Ward’s method involves looking at the distances between all pairs and averages all of these distances. To identify the optimal number of clusters, and to assess the robustness of the clustering results, we computed the silhouette scores and gap statistic for different numbers of clusters from two to five [25]. To confirm unsupervised clustering results, we used *t*-distributed stochastic neighborhood embedding (*t*-SNE) [26], a powerful dimensionality reduction method. The *t*-SNE method captures the variance in the data by attempting to preserve the distances between data points from high to low dimensions without any prior assumptions about the data distribution.

### Classification using a Bayesian classifier

We constructed a classifier, where a set of predictors consists of 26 pathways, using a naive Bayes machine learning algorithm [27]. For training the classifier, we used the pathway enrichment scores and subgroup labels of the result of the agglomerative hierarchical clustering process. We controlled overfitting in modeling by using 10-fold cross-validation and applied the 26-pathway classifier to assign subgroups to the new samples.

### Key driver analysis

To predict genes that modulate the regulatory state of the disease module, we employed key driver analysis (KDA), an algorithm that mathematically identifies causal modulators of the regulatory state of functionally relevant gene groups [7, 8, 28, 29]. Bayesian networks are directed acyclic graphs in which the edges of the graph are defined by conditional probabilities that characterize the distribution of states of each node given the state of its parents. The network topology defines a partitioned joint probability distribution over all nodes in a network, such that the probability distribution of states of a node depends only on the states of its parent nodes [7]. KDA to identify key driver genes (KDGs) takes as input a set of genes (*G*) and a directed gene network (*N*; a Bayesian network). The objective is to identify the key regulators for the gene sets with respect to the given network. KDA first generates a subnetwork *NG*, defined as the set of nodes in *N* that are no more than *h* layers away from the nodes in *G*, and then searches the *h*-layer neighborhood (*h* = 1,…, *H*) for each gene in *NG* (HLN_*g*,*h*_) for the optimal h*, such that

$$ {\mathrm{ES}}_h\ast =\max \left({\mathrm{ES}}_{h,g}\right)\forall g\in {N}_{g,h}\in \left\{1,,\dots,, H\right\} $$

where ES_*h*,*g*_ is the computed enrichment statistic for HLN_*g*,*h*_. A node becomes a candidate driver if its HLN is significantly enriched for the nodes in *G*. Candidate drivers without any parent node (i.e., root nodes in directed networks) are designated as global drivers and the rest are local drivers. The statistical significance of a key driver for a given gene set in a particular Bayesian network is determined by Fisher’s exact test which assesses the enrichment of the genes in the candidate key driver’s network neighborhood. Bonferroni-corrected *P* < 0.01 was used to determine key drivers. Functional classification of the genes was detected by DAVID Bioinformatics resources [30].

### Statistical analysis

For continuous distributed data, between-group comparisons were performed using the unpaired *t* test or Mann-Whitney *U* test. Categorical or dichotomous variables were compared using the chi-squared test or Fisher’s exact test. Correlation analysis between two variables was carried out using Pearson’s correlation coefficient. All analyses were conducted in R (version 3.6.0, The R Project for Statistical Computing, www.r-project.org).

## Results

### Differentially expressed genes and their protein-protein interaction network

**Fig. 2:**
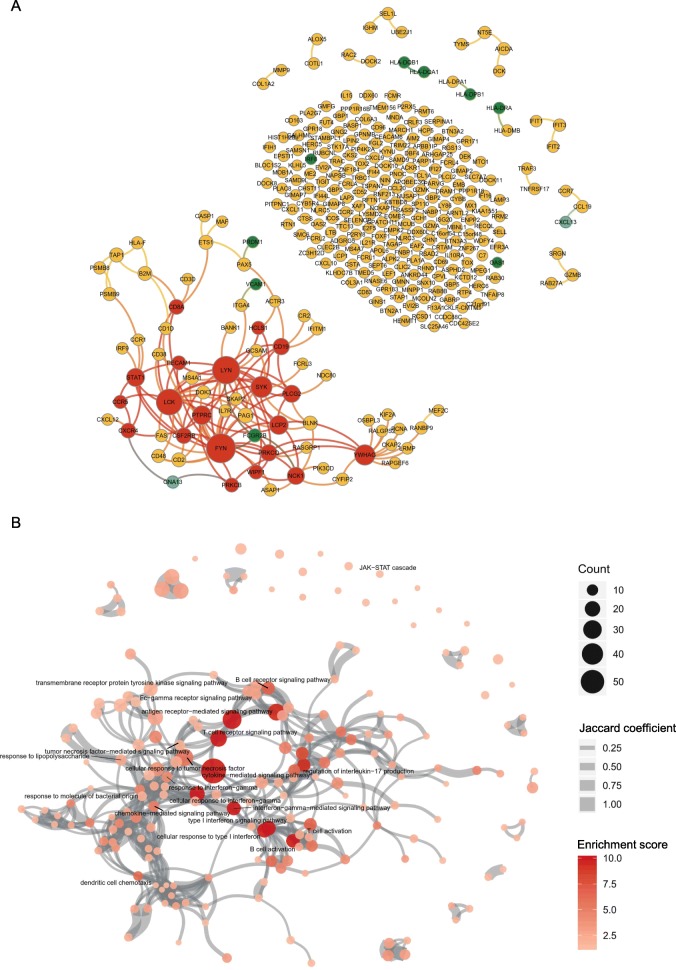
Differentially expressed genes and their functional networks. **a** Protein-protein interaction network of upregulated DEGs. Informative genes are colored and identified in the right-side table. **b** Functional enrichment map from the functional enrichment analysis using the Enrichr tool (https://amp.pharm.mssm.edu/Enrichr3/). Nodes represent gene ontology–biological process (GO-BP) gene sets, and GO-BPs of interest are labeled. Their color intensity and size are proportional to the enrichment score and the gene size, respectively. The edge thickness represents the degree of overlap between gene sets, and only edges with a Jaccard similarity coefficient larger than 0.25 are visualized. See Additional file 1: Figure S2 for the full node labels

An overview of our study design, including sample processing and the integrative network-based approach, is shown in Fig. 1. A list of DEGs was attained by the comparison of gene expression profiles of the salivary gland from SjS patients with those from normal healthy controls (NCs). A total of 310 upregulated DEGs were identified in SjS (Additional file 2). Because identification of central attractors in the gene and protein network can provide clues about novel disease-associated genes with high priority or hidden targets for further experimentation, we constructed a protein-protein interaction network for SjS (Fig. 2a). We identified 156 interactions of the 310 DEGs, and 211 genes were isolated without a direct link. The network included nine genetic risk factors (*FCGR2B*, *HLA-DPB1*, *HLA-DQA1*, *HLA-DQB1*, *HLA-DRA*, *IRF8*, *OAS1*, *PRDM1*, and *VCAM1*) [31–33] and two promising biomarkers (*CXCL13* and *GNA13*) [34, 35]. The largest connect component (LCC), also known as the giant component, is a connected component of a network that contains a significant proportion of the entire nodes in the network [36, 37]. The LCC is usually the most complex part of the network; it represents a core that sustains the whole network [38]. LCC of the network consisted of 69 genes and 20 genes were ranked as hub molecules based on centrality analysis. The top five hub molecules in order of degree were *FYN*, *LYN*, *LCK*, *SYN*, and *YWHAG*.

### Enriched biological processes

We performed functional enrichment analysis for the DEGs using the Enrichr tool [19], from which 194 gene ontology (GO)–biological process terms were identified (Fig. 2b and Additional file 1: Figure S2). Type I and II IFN-related (type I IFN signaling pathway, cellular response to IFN-γ, cellular response to type I IFN, IFN-γ-mediated signaling pathway), B cell-related (B cell receptor (BCR) signaling pathway, B cell activation), and other major immune response-related (cytokine-mediated signaling pathway, dendritic cell chemotaxis, antigen receptor-mediated signaling pathway) processes were predominantly enriched (Fig. 2b and Additional file 1: Figure S2). This result was in concordance with the current concept of salivary gland pathophysiology in SjS [1, 2, 4, 5]. The LCC of the protein-protein interaction network was notably enriched for B cell activation (*P* = 6.29 × 10^−13^), BCR signaling pathway (*P* = 1.33 × 10^−8^), regulation of BCR signaling pathway (*P* = 2.90 × 10^−7^), Fc-γ receptor signaling pathway (*P* = 8.67 × 10^−8^), and the antigen receptor-mediated signaling pathway (*P* = 1.46 × 10^−14^).

### Enriched pathways describing SjS pathophysiology and subgrouping

**Fig. 3:**
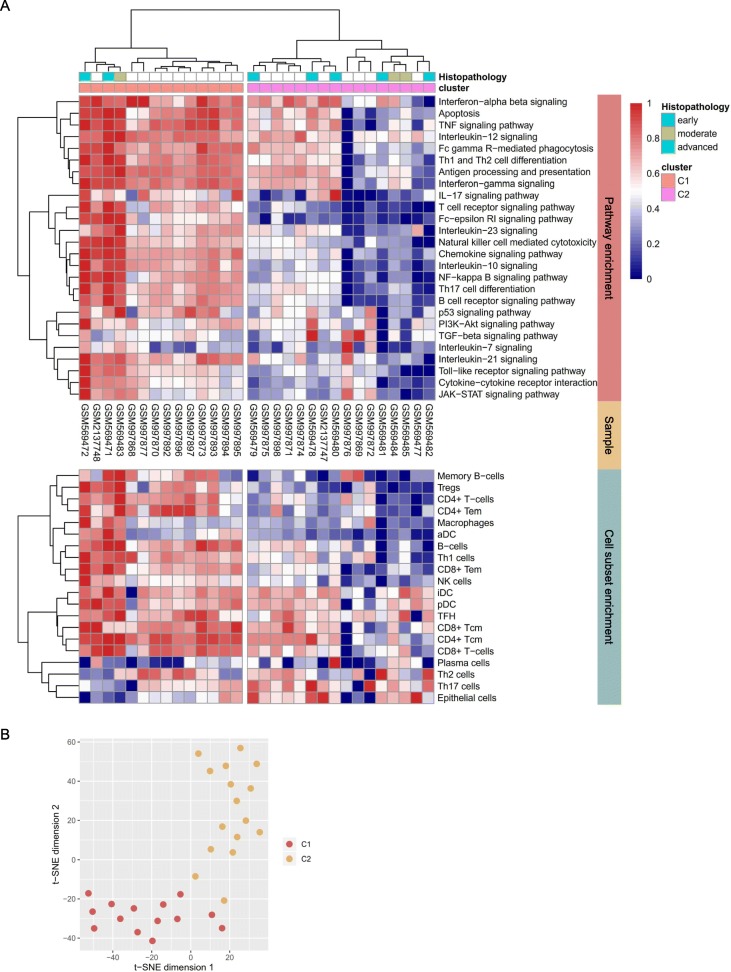
Identification of SjS subgroups according to pathway-driven enrichment profiles. **a** Heatmaps and hierarchical clustering of gene set enrichment scores by pathways and cell subsets. A matrix of pathway-driven enrichment score was clustered by agglomerative hierarchical clustering and a heatmap of cell subset-driven enrichment scores was matched side-by-side. The histopathological grades for ten samples are tagged on top of the heatmap. **b** *t*-SNE reduces the dimensions of a multivariate dataset. Each data point is assigned a location in a two-dimensional map to illustrate potential clusters of neighboring samples, which contain similar pathway activity patterns. aDC, activated dendritic cells; cm, cytotoxic memory; em, effector memory; iDC, immature dendritic cells; pDC, plasmocytoid dendritic cells

Genes, proteins, and other chemical compounds in a living organism rarely act in isolation, but work cooperatively to perform certain biological functions. In the same vein, disease is the summed result of aberrant activation of common pathways through dysregulated genes and aggregated activity of compounds [39]. The advantage of pathway-based analysis has been previously demonstrated in clinical stratification for inflammatory disease and cancer research [11, 40–42]. We curated 26 pathways or processes representing SjS pathophysiology from the literature [1, 2, 4, 5] and computed a pathway enrichment score for the gene sets from the KEGG and Reactome databases for each sample using a single sample version of GSEA (Additional file 3) [43, 44]. We assessed whether SjS patients could be categorized into subgroups based on their pathway enrichment profiles through agglomerative hierarchical clustering [24]. To identify the optimal number of clusters, and to assess the robustness of the clustering results, we computed the silhouette scores and gap statistic for different numbers of clusters from two to five [25], and found that two clusters most optimally represented the data (Fig. 3a and Additional file 1: Figure S3). In a between-cluster comparison analysis, enrichment scores of all the pathways except the transforming growth factor (TGF)-β and IL-7 signaling pathways significantly differed (all *P* < 0.05) and segregation of the SjS subgroups was reproduced by *t*-SNE analysis (Fig. 3b).

Cluster 1 showed strong enrichment for most of the pathways, whereas, in cluster 2, a limited number of pathways such as the IFN-α,β, IFN-γ, TNF, and IL-12 signaling pathways were moderately enriched in a subset of the samples (Fig. 3a, upper panel). One of the datasets, GSE23117, included ten salivary gland samples annotated with histopathological scores: five early (one focus), three moderate (two to three foci), and two advanced (diffuse infiltration with partial destruction of acinar tissue) [45]. Considering the histopathological status of the samples tagged on top of the clustering heatmap, cluster 1 favored moderate to advanced status, while cluster 2 inclined toward early to moderate status (Fig. 3a).

To characterize the cell types responsible for gene expression differences among the salivary gland samples, we applied xCell software, the machine learning framework to estimate cell type enrichment [23]. Cluster 1 was more enriched with B cells, CD4^+^ T cells, CD8^+^ T cells, follicular helper T(T_FH_) cells, Th1 cells, regulatory T(Treg) cells, natural killer(NK) cells, and macrophages, while cluster 2 was enriched with epithelial cells (*P* < 0.05) (Fig. 3a, lower panel). Enrichment of immature and plasmacytoid dendritic cells, plasma cells, and Th2 and Th17 cells were not different between the two clusters (*P* > 0.05).

### Evolution of pathways and cell subset enrichment in an SjS-like mouse model

**Fig. 4:**
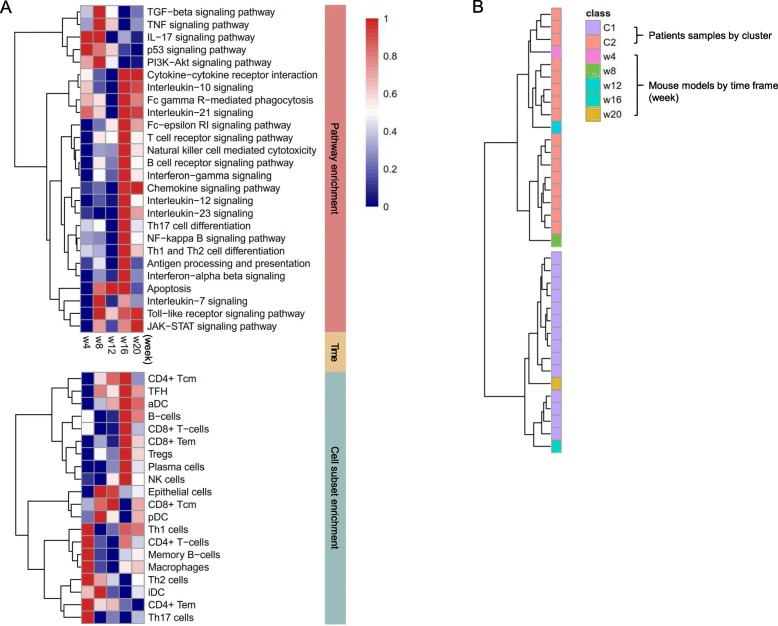
Evolutional patterns of pathway- and cell subset-driven enrichment score in a SjS-like mouse model. **a** A heatmap of the averaged pathway- and cell subset-driven enrichment scores by time points. **b** Clustering of integrated human and mouse pathway-driven enrichment profiles. The left dendrogram shows the organization of the molecular subsets of SjS. Pathway-driven enrichment profiles from the mouse model are interspersed among the human subsets

To verify the transitional change of salivary gland molecular signatures in SjS, we imported GSE15640, the salivary gland microarray datasets of five equally spaced time points in a C57BL/6.NOD-*Aec1Aec2* mouse [46], which is a good model reproducing the immunopathological abnormalities and clinical phenotypes of SjS [47, 48]. Pathway- and cell subset-driven enrichment scores by time frame are summarized in Fig. 4a. In the earlier phase (week 4 or 8), IL-7 and IL-17 signaling pathways were active and Th1, Th2, and Th17 cells were highly enriched. The molecular signature of epithelial cells was increased at weeks 8 and 12. The enrichment score of most of the SjS-relevant pathways and key immune cells (B cells, T_FH_ cells, and NK cells) peaked at week 16 and weakened by week 20. When compared with the patient’ samples by hierarchical clustering, the earlier phase (weeks 4, 8, and 12) was similar to cluster 2, while the later phase (week 16 and 20) bore a close parallel to cluster 1 (Fig. 4b and Additional file 1: Figure S4).

### Association of pathway-driven subgroups with clinical index

**Fig. 5:**
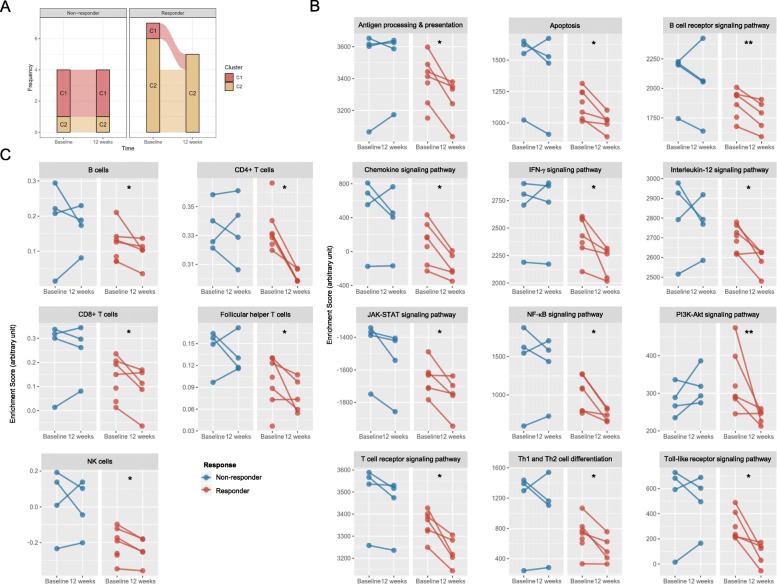
Temporal changes of molecular traits in SjS patients receiving rituximab therapy. **a** Temporal change of the cluster classification at baseline and 12 weeks later by treatment response. C1 and C2 indicate clusters 1 and 2, respectively, and the samples were classified according to the modeled Bayesian classifier. **b** Temporal change of the enrichment scores of the main SjS-relevant pathways at baseline and 12 weeks later by treatment response. **c** Temporal change of the enrichment scores of the main cell subsets at baseline and 12 weeks later by treatment response. Samples from the same patient are linked by a line. The red- and blue-colored dots indicate responders and non-responders, respectively. The difference between the two time points was tested by paired *t* test. *P < 0.05; **P < 0.01

The microarray dataset E-MEXP-1883 includes 11 paired samples of baseline and 12-week follow-up patients from an open-label trial of rituximab therapy in patients with SjS [49]. Patients were considered responders to rituximab if they had a ≥ 30% improvement between weeks 0 and 12 in the value on at least three of the four visual analog scales. A 26-pathway classifier was developed using a naive Bayes machine learning algorithm to predict the clusters for the new samples corresponding to the above result. The classifier successfully categorized the samples into two clusters. The evolution of the samples by treatment response and cluster is summarized in Fig. 5a. Most of the responders (85.7%) were cluster 2 and all converted into cluster 2 at week 12 after rituximab therapy. To investigate the change of SjS-relevant pathways and cell subsets, pathway- and cell subset-driven enrichment scores were computed (Fig. 5b, c). In the responders, the main SjS-relevant pathways, including BCR, chemokine, IFN-γ, IL-12, and T cell-receptor signaling pathways, were significantly downregulated at week 12 after rituximab therapy (all *P* < 0.05), and the key cellular components (B cells, CD4^+^ T cells, CD8^+^ T cells, T_FH_ cells, and NK cells) also diminished (all *P* < 0.05). In contrast, the non-responders had much higher signals for the pathways or cell subsets at baseline compared with the responders or showed a worsening tendency. Taken together, it is supposed that cluster 1 represents the advanced status of high-grade inflammation, while cluster 2 is the early or regressed status of low-grade inflammation.

### Identifying causal key regulators of the disease module

**Fig. 6:**
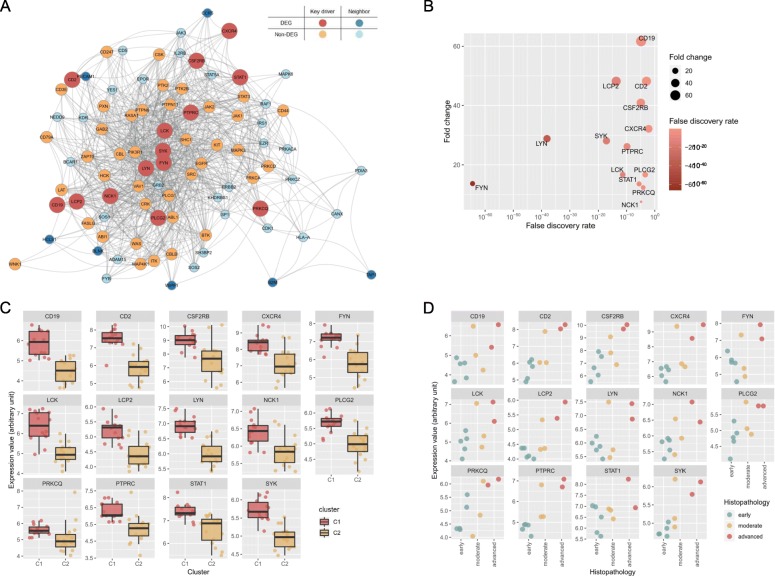
Key driver analysis. **a** Probabilistic causal gene network projection and key driver analysis identifies causal regulators of the core SjS subnetwork. Key driver genes (KDGs) and their neighbors are distinguished by color. **b** Fold change and false discovery rate of the differentially expressed KDGs. **c** Expression levels of the KDGs by cluster classification. Expression levels of all KDGs (*P* < 0.001) except for *PRKCQ* (*P* = 0.058) were significantly different between the two clusters by *t* test. **d** Expression levels of the KDGs by the histopathological scores. Samples were categorized as early (1 focus), moderate (2–3 foci), and advanced (diffuse infiltration with partial destruction of acinar tissue) by their histopathology

Elucidating the connectivity structure within the disease module can lead to the identification of key driver genes (KDGs) that are predicted to modulate the regulatory state of the module, and will be of high interest to prioritize as causal to disease development and progression. We constructed a Bayesian network by projecting the genes from the LCC onto the human interactome and employed key driver analysis (KDA), an algorithm that mathematically identifies causal modulators of the regulatory state of functionally relevant gene groups to predict gene that modulate the regulatory state of the SjS core module [7, 8, 28, 29]. We identified 14 differentially expressed KDGs (Fig. 6a, b). In gene functional classification analysis using DAVID bioinformatics resources [30], SYK tyrosine kinase and members of the Src family kinase (FYN, LCK, and LYN) were the key mediators in regulating signal transduction concerning the BCR, T cell-receptor signaling pathways, and/or NK cell-mediated cytotoxicity. The expression values of the KDGs were remarkably higher in cluster 1 compared with cluster 2 (Fig. 6c) and displayed a rising tendency as the histopathological score of the salivary glands increased (Fig. 6d). We identified that the BCR signaling pathway and B cell activation were the main processes of the core subnetwork, the LCC, in SjS (Fig. 2a). The expression values of the KDGs were also closely correlated with the enrichment scores of the BCR signaling pathway and B cell activation (Additional file 1: Figure S5). Leading-edge genes in a GSEA are those that contribute most to the enrichment of a particular gene set and include the most significantly upregulated genes in a given gene set [20]. *BTK*, *CR2*, *BLINK*, *PRKCB*, *PIK3CD*, and *PLCG2* were the leading-edge genes shared by both the BCR signaling pathway and B cell activation (Additional file 1: Figures S6 and S7).

## Discussion

In the present study, we collected salivary gland transcriptomic profiles from patients with SjS and an SjS-like mouse model. We carried out an integrative analysis to understand differential expression patterns by histopathologic index or treatment response in terms of the pathways and cell subsets and to identify key drivers and molecules that may serve as effective targets for therapeutic intervention. The core processes of the DEG network in SjS were the BCR signaling pathway and B cell activation, supported by activated T cells and various kinds of cytokines. Unsupervised cluster analysis of the SjS transcriptomic profiles resulted in two subgroups of SjS patients with distinct activities of the relevant pathways, which had a positive relationship with histopathology scores and showed differing responses to rituximab therapy. To pinpoint key regulators, we projected the SjS core gene set onto the human interactome and identified KDGs. These KDGs appear to be essential linkers or signaling mediators downstream of the SjS core biological processes.

Although many immunomodulatory therapies or biologics have been trialed in SjS, the primary efficacy endpoint has not been met and these treatments have not been proven effective [50]. This could be explained not only by a true lack of efficacy, but also by the heterogeneity of the patients’ disease status. SjS is a slowly progressing chronic autoimmune disease and patients present with extremely variable symptoms and inflammatory levels of the salivary glands. We constructed pathway-driven enrichment score profiles across the patients, and these were optimally separated into two clusters by their similarity. Cluster 1 was a high-grade inflammatory status enriched in a number of the main immune cells, especially for B cells and Th1 cells. In contrast, cluster 2 was a low-grade inflammatory condition with a weak signature for immune cells except for epithelial cells and Th17 cells. This result matched with the histopathological scores and the evolutional change of gene expression at salivary glands in an SjS-like mouse model. It is noteworthy that the clusters determined the response to rituximab therapy. Patients classified as cluster 2 showed better outcomes and the main signaling pathways and immune cell activities were effectively downregulated, which was in concordance with previous results [51–53]. However, patients in cluster 1 did not, and poor responders also existed in the prospective clinical trials [53]. It could be suggested that an early stage of SjS with suboptimally activated B cells, as in cluster 2, can be subdued by B cell depletion therapy, while the advanced subgroup with fully activated B cells with assistance from other immune cells, as in cluster 1, cannot be adequately controlled by rituximab and other approaches would be required. Future clinical trials can consider this categorization using gene expression profiling and differential analysis for treatment response. This approach can also be used in a clinical setting to determine whether a certain group of patients are more responsive to the investigational drug than other types of patients.

B cell hyperactivity was the key process in the core subnetwork of SjS and cluster 1 showed much stronger signatures for B cells and the associated pathological pathways compared with cluster 2. Bayesian networks have been successfully used to derive causal influences among biological signaling molecules [54, 55]; moreover, they have been successfully applied in the discovery of key regulators in various diseases such as inflammatory bowel disease and Alzheimer’s disease [7, 8, 28, 29]. We constructed differentially expressed and probabilistic causal gene networks to model molecular interactions and causal gene relationships, and applied Bayesian networks-based KDA to identify and prioritize the key drivers of SjS. The 14 KDGs were distinctively expressed by cluster and had a positive relationship with the histopathology scores, representing their leading role in the immunoinflammatory response of SjS. *SYK*, *LYN*, *NCK1*, and *PLCG2* are the key mediators regulating signal transduction of the BCR signaling pathway [56, 57] and *CD19*, *FYN*, and *LCK* are also linked to this pathway through the PI3K–Akt pathway [57–59]. In addition, *BTK*, *BLINK*, and *PIK3CD*, the differentially expressed leading-edge genes of the BCR signaling pathway, were situated in the middle of the KDG network to effectively perturb the KDGs. These results are reminiscent of B cell malignancies such as chronic lymphocytic leukemia [56, 60] and could provide a clue to the cause of unsatisfactory SjS treatment. In chronic lymphocytic leukemia, selective inhibitors against BTK and PI3KCD are used for patients unsuitable or refractory to the rituximab-based chemotherapy regimen [60]. In particular, we found that PIK3CD was a DEG in the SjS salivary gland. Its product, PI3Kδ, critically regulates a number of signaling pathways driven by receptors including BCR, Fc-γ receptor, and CXCR4, and functions to integrate and transduce these signals from the microenvironment, thus promoting B cell proliferation, growth, survival, adhesion, and homing [57]. In a recent study by Nayar et al., the administration of PI3Kδ-selective inhibitors showed significant therapeutic efficacy in a murine model of focal sialoadenitis by reducing cytokine production and accumulation of lymphocytes within the glands [61]. Taken together, a specific therapy engineered to interrupt the BCR signaling pathway would be promising for achieving better outcomes, especially for patients with severe inflammation and lymphocytic infiltration in the salivary glands.

There are some limitations to address in this study. First, the number of patient’ samples was not large enough, although we gathered all the available datasets. The accumulation of more data in the future could facilitate more precise subgrouping and analysis. Second, we did not address the association of each SjS subgroup with other clinical factors, such as autoantibodies and disease activity indices, because of a lack of complete annotation for those parameters. Third, minority signatures by specific processes or cell subsets might have been diluted because the gene expression signature was at the tissue level and on a wide spectrum across the patients.

## Conclusion

SjS is a major medical challenge with a high unmet need. In this study, we comprehensively profiled salivary gland transcriptomic changes in SjS individuals. By adopting an integrative, data-driven approach, we demonstrated the breadth of cellular and mechanistic signatures in SjS, separated the patients into two subtypes with distinct molecular traits and treatment responses, and suggested the promising molecular targets based on these subtypes. This combination of findings is useful for ensuring better targeting of B cell hyperactivity and concomitantly better selection of patients most likely to benefit from investigational drugs, potentially enabling more personalized therapy in the future.

## Supplementary information

**Additional file 1. Figure S1.** Principal component analysis on the merged gene expression profiles of salivary gland before and after normalization and batch correction. **Figure S2.** Functional enrichment map for up-regulated DEG. **Figure S3.** Identification of the optimal number of clusters. **Figure S4.** Hierarchical clustering of pathway enrichment profiles from patients with SjS and SjS-like mouse models. **Figure S5.** Correlation between two key pathways enrichment score and KDGs expression values. **Figure S6.** Enrichment and leading-edge genes of the B cell receptor signaling pathway and B cell activation. **Figure S7.** Details on the KDGs and the leading edge genes from the B cell receptor signaling pathway and B cell activation.

**Additional file 2.** A full list of differentially expressed genes (up-regulated and down-regulated).

**Additional file 3.** A matrix of enrichment scores by single-sample gene-set enrichment analysis Sheet #1: Data from patients with SjS (GSE7307, GSE23117, GSE40611, and GSE80805). Sheet #2: Data from SjS patients treated with rituximab (E-MEXP-1883).

## Data Availability

All the processed data were included in the current study.

## Abbreviations

BCR: B cell receptor
BAFF: B cell-activating factor
DEGs: Differentially expressed genes
TFH: Follicular helper T
GO: Gene ontology
GSEA: Gene set enrichment analysis
IFN: Interferon
IL: Interleukin
KDA: Key driver analysis
KDGs: Key driver genes
LCC: Largest connected component
NC: Normal healthy control
SGECs: Salivary gland epithelial cells
SjS: Sjögren’s syndrome
*t*-SNE: *t*-Distributed stochastic neighborhood embedding
TLR: Toll-like receptor
TGF: Transforming growth factor

## References

[1] Brito-Zeron P, Baldini C, Bootsma H, Bowman SJ, Jonsson R, Mariette X, Sivils K, Theander E, Tzioufas A, Ramos-Casals M (2016). Sjogren syndrome. Nature Reviews Disease Primers.

[2] Mariette X, Criswell LA (2018). Primary Sjogren’s syndrome. N Engl J Med.

[3] Felten R, Scher F, Sibilia J, Gottenberg JE, Arnaud L (2019). The pipeline of targeted therapies under clinical development for primary Sjogren's syndrome: a systematic review of trials. Autoimmun Rev.

[4] Kwok SK, Lee J, Yu D, Kang KY, Cho ML, Kim HR, Ju JH, Lee SH, Park SH, Kim HY (2015). A pathogenetic role for IL-21 in primary Sjogren syndrome. Nat Rev Rheumatol.

[5] Nocturne G, Mariette X (2013). Advances in understanding the pathogenesis of primary Sjogren’s syndrome. Nat Rev Rheumatol.

[6] Parikshak NN, Luo R, Zhang A, Won H, Lowe JK, Chandran V, Horvath S, Geschwind DH (2013). Integrative functional genomic analyses implicate specific molecular pathways and circuits in autism. Cell.

[7] Zhang B, Gaiteri C, Bodea LG, Wang Z, McElwee J, Podtelezhnikov AA, Zhang C, Xie T, Tran L, Dobrin R (2013). Integrated systems approach identifies genetic nodes and networks in late-onset Alzheimer’s disease. Cell.

[8] Peters LA, Perrigoue J, Mortha A, Iuga A, Song WM, Neiman EM, Llewellyn SR, Di Narzo A, Kidd BA, Telesco SE (2017). A functional genomics predictive network model identifies regulators of inflammatory bowel disease. Nat Genet.

[9] Squair JW, Tigchelaar S, Moon KM, Liu J, Tetzlaff W, Kwon BK, Krassioukov AV, West CR, Foster LJ, Skinnider MA (2018). Integrated systems analysis reveals conserved gene networks underlying response to spinal cord injury. Elife.

[10] Kim KJ, Kim M, Adamopoulos IE, Tagkopoulos I (2019). Compendium of synovial signatures identifies pathologic characteristics for predicting treatment response in rheumatoid arthritis patients. Clin Immunol.

[11] Moon SJ, Bae JM, Park KS, Tagkopoulos I, Kim KJ (2019). Compendium of skin molecular signatures identifies key pathological features associated with fibrosis in systemic sclerosis. Ann Rheum Dis.

[12] Vitali C, Bombardieri S, Jonsson R, Moutsopoulos HM, Alexander EL, Carsons SE, Daniels TE, Fox PC, Fox RI, Kassan SS (2002). Classification criteria for Sjogren's syndrome: a revised version of the European criteria proposed by the American-European Consensus Group. Ann Rheum Dis.

[13] Shiboski SC, Shiboski CH, Criswell L, Baer A, Challacombe S, Lanfranchi H, Schiodt M, Umehara H, Vivino F, Zhao Y (2012). American College of Rheumatology classification criteria for Sjogren’s syndrome: a data-driven, expert consensus approach in the Sjogren’s International Collaborative Clinical Alliance cohort. Arthritis Care Res (Hoboken).

[14] Chen C, Grennan K, Badner J, Zhang D, Gershon E, Jin L, Liu C (2011). Removing batch effects in analysis of expression microarray data: an evaluation of six batch adjustment methods. PLoS One.

[15] Muller C, Schillert A, Rothemeier C, Tregouet DA, Proust C, Binder H, Pfeiffer N, Beutel M, Lackner KJ, Schnabel RB (2016). Removing batch effects from longitudinal gene expression - quantile normalization plus ComBat as best approach for microarray transcriptome data. PLoS One.

[16] Ritchie ME, Phipson B, Wu D, Hu Y, Law CW, Shi W, Smyth GK (2015). limma powers differential expression analyses for RNA-sequencing and microarray studies. Nucleic Acids Res.

[17] Menche J., Sharma A., Kitsak M., Ghiassian S. D., Vidal M., Loscalzo J., Barabasi A.-L. (2015). Uncovering disease-disease relationships through the incomplete interactome. Science.

[18] Koschutzki D, Schreiber F (2008). Centrality analysis methods for biological networks and their application to gene regulatory networks. Gene Regulation Systems Biol.

[19] Kuleshov MV, Jones MR, Rouillard AD, Fernandez NF, Duan Q, Wang Z, Koplev S, Jenkins SL, Jagodnik KM, Lachmann A (2016). Enrichr: a comprehensive gene set enrichment analysis web server 2016 update. Nucleic Acids Res.

[20] Subramanian A, Tamayo P, Mootha VK, Mukherjee S, Ebert BL, Gillette MA, Paulovich A, Pomeroy SL, Golub TR, Lander ES (2005). Gene set enrichment analysis: a knowledge-based approach for interpreting genome-wide expression profiles. Proc Natl Acad Sci U S A.

[21] Merico D, Isserlin R, Stueker O, Emili A, Bader GD (2010). Enrichment map: a network-based method for gene-set enrichment visualization and interpretation. PLoS One.

[22] Barbie DA, Tamayo P, Boehm JS, Kim SY, Moody SE, Dunn IF, Schinzel AC, Sandy P, Meylan E, Scholl C (2009). Systematic RNA interference reveals that oncogenic KRAS-driven cancers require TBK1. Nature.

[23] Aran D, Hu Z, Butte AJ (2017). xCell: digitally portraying the tissue cellular heterogeneity landscape. Genome Biol.

[24] Murtagh F, Legendre P (2014). Ward’s hierarchical agglomerative clustering method: which algorithms implement Ward’s criterion?. J Classif.

[25] Mirkin Boris (2011). Choosing the number of clusters. Wiley Interdisciplinary Reviews: Data Mining and Knowledge Discovery.

[26] Maaten LVD, Hinton GE (2008). Visualizing data using t-SNE. J Machine Learning Res.

[27] Domingos P, Pazzani M (1997). On the optimality of the simple Bayesian classifier under zero-one loss. Mach Learn.

[28] Shu L, Zhao Y, Kurt Z, Byars SG, Tukiainen T, Kettunen J, Orozco LD, Pellegrini M, Lusis AJ, Ripatti S (2016). Mergeomics: multidimensional data integration to identify pathogenic perturbations to biological systems. BMC Genomics.

[29] Watson CT, Cohain AT, Griffin RS, Chun Y, Grishin A, Hacyznska H, Hoffman GE, Beckmann ND, Shah H, Dawson P (2017). Integrative transcriptomic analysis reveals key drivers of acute peanut allergic reactions. Nat Commun.

[30] da Huang W, Sherman BT, Lempicki RA (2009). Systematic and integrative analysis of large gene lists using DAVID bioinformatics resources. Nat Protoc.

[31] Lessard CJ, Li H, Adrianto I, Ice JA, Rasmussen A, Grundahl KM, Kelly JA, Dozmorov MG, Miceli-Richard C, Bowman S (2013). Variants at multiple loci implicated in both innate and adaptive immune responses are associated with Sjogren’s syndrome. Nat Genet.

[32] Li Y, Zhang K, Chen H, Sun F, Xu J, Wu Z, Li P, Zhang L, Du Y, Luan H (2013). A genome-wide association study in Han Chinese identifies a susceptibility locus for primary Sjogren's syndrome at 7q11.23. Nat Genet.

[33] Teos LY, Alevizos I (2017). Genetics of Sjogren’s syndrome. Clin Immunol.

[34] Kramer JM, Klimatcheva E, Rothstein TL (2013). CXCL13 is elevated in Sjogren’s syndrome in mice and humans and is implicated in disease pathogenesis. J Leukoc Biol.

[35] Aqrawi LA, Galtung HK, Vestad B, Ovstebo R, Thiede B, Rusthen S, Young A, Guerreiro EM, Utheim TP, Chen X (2017). Identification of potential saliva and tear biomarkers in primary Sjogren’s syndrome, utilising the extraction of extracellular vesicles and proteomics analysis. Arthritis Res Therapy.

[36] Barabási AL, PÃ3sfai MÃ. Network Science. Cambridge: Cambridge University Press; 2016.

[37] Ashtiani M, Salehzadeh-Yazdi A, Razaghi-Moghadam Z, Hennig H, Wolkenhauer O, Mirzaie M, Jafari M (2018). A systematic survey of centrality measures for protein-protein interaction networks. BMC Syst Biol.

[38] Callaway DS, Newman MEJ, Strogatz SH, Watts DJ (2000). Network robustness and fragility: percolation on random graphs. Phys Rev Lett.

[39] Kim YA, Wuchty S, Przytycka TM (2011). Identifying causal genes and dysregulated pathways in complex diseases. PLoS Comput Biol.

[40] Drier Y, Sheffer M, Domany E (2013). Pathway-based personalized analysis of cancer. Proc Natl Acad Sci U S A.

[41] You S, Knudsen BS, Erho N, Alshalalfa M, Takhar M, Al-Deen Ashab H, Davicioni E, Karnes RJ, Klein EA, Den RB (2016). Integrated classification of prostate cancer reveals a novel luminal subtype with poor outcome. Cancer Res.

[42] Weiser M, Simon JM, Kochar B, Tovar A, Israel JW, Robinson A, Gipson GR, Schaner MS, Herfarth HH, Sartor RB (2018). Molecular classification of Crohn’s disease reveals two clinically relevant subtypes. Gut.

[43] Fabregat A, Sidiropoulos K, Garapati P, Gillespie M, Hausmann K, Haw R, Jassal B, Jupe S, Korninger F, McKay S (2016). The Reactome pathway knowledgebase. Nucleic Acids Res.

[44] Kanehisa M, Furumichi M, Tanabe M, Sato Y, Morishima K (2017). KEGG: new perspectives on genomes, pathways, diseases and drugs. Nucleic Acids Res.

[45] Greenwell-Wild T, Moutsopoulos NM, Gliozzi M, Kapsogeorgou E, Rangel Z, Munson PJ, Moutsopoulos HM, Wahl SM (2011). Chitinases in the salivary glands and circulation of patients with Sjogren’s syndrome: macrophage harbingers of disease severity. Arthritis Rheum.

[46] Nguyen CQ, Sharma A, Lee BH, She JX, McIndoe RA, Peck AB (2009). Differential gene expression in the salivary gland during development and onset of xerostomia in Sjogren’s syndrome-like disease of the C57BL/6.NOD-Aec1Aec2 mouse. Arthritis Res Ther.

[47] Donate A, Voigt A, Nguyen CQ (2014). The value of animal models to study immunopathology of primary human Sjogren’s syndrome symptoms. Expert Rev Clin Immunol.

[48] Park YS, Gauna AE, Cha S (2015). Mouse models of primary Sjogren’s syndrome. Curr Pharm Des.

[49] Devauchelle-Pensec V, Cagnard N, Pers JO, Youinou P, Saraux A, Chiocchia G (2010). Gene expression profile in the salivary glands of primary Sjogren’s syndrome patients before and after treatment with rituximab. Arthritis Rheum.

[50] Saraux A, Pers JO, Devauchelle-Pensec V (2016). Treatment of primary Sjogren syndrome. Nat Rev Rheumatol.

[51] Delli K, Haacke EA, Kroese FG, Pollard RP, Ihrler S, van der Vegt B, Vissink A, Bootsma H, Spijkervet FK (2016). Towards personalised treatment in primary Sjogren’s syndrome: baseline parotid histopathology predicts responsiveness to rituximab treatment. Ann Rheum Dis.

[52] Carubbi F, Cipriani P, Marrelli A, Benedetto P, Ruscitti P, Berardicurti O, Pantano I, Liakouli V, Alvaro S, Alunno A (2013). Efficacy and safety of rituximab treatment in early primary Sjogren’s syndrome: a prospective, multi-center, follow-up study. Arthritis Res Ther.

[53] Verstappen GM, van Nimwegen JF, Vissink A, Kroese FGM, Bootsma H (2017). The value of rituximab treatment in primary Sjogren’s syndrome. Clin Immunol.

[54] Sachs K, Perez O, Pe'er D, Lauffenburger DA, Nolan GP (2005). Causal protein-signaling networks derived from multiparameter single-cell data. Science (New York, NY).

[55] Pe'er D (2005). Bayesian network analysis of signaling networks: a primer. Sci STKE.

[56] Burger JA, Wiestner A (2018). Targeting B cell receptor signalling in cancer: preclinical and clinical advances. Nat Rev Cancer.

[57] Yang Q, Modi P, Newcomb T, Queva C, Gandhi V (2015). Idelalisib: first-in-class PI3K Delta inhibitor for the treatment of chronic lymphocytic leukemia, small lymphocytic leukemia, and follicular lymphoma. Clin Cancer Res.

[58] Xu Y, Huntington ND, Harder KW, Nandurkar H, Hibbs ML, Tarlinton DM (2012). Phosphatidylinositol-3 kinase activity in B cells is negatively regulated by Lyn tyrosine kinase. Immunol Cell Biol.

[59] Talab F, Allen JC, Thompson V, Lin K, Slupsky JR (2013). LCK is an important mediator of B-cell receptor signaling in chronic lymphocytic leukemia cells. Mol Cancer Res.

[60] Hallek M, Shanafelt TD, Eichhorst B (2018). Chronic lymphocytic leukaemia. Lancet.

[61] Nayar S, Campos J, Smith CG, Iannizzotto V, Gardner DH, Colafrancesco S, Pipi E, Kollert F, Hunter KJ, Brewer C (2019). Phosphatidylinositol 3-kinase delta pathway: a novel therapeutic target for Sjogren’s syndrome. Ann Rheum Dis.

