# Supplementary material for: Integrated systems analysis of salivary gland transcriptomics reveals key molecular networks in Sjögren’s syndrome
Source: Arthritis Res Ther. 2019 Dec 19;21:294. doi: 10.1186/s13075-019-2082-9 (PMC6921432; doi:10.1186/s13075-019-2082-9)
Supplement: Supplementary file 1 — Additional file 1. Figure S1. Principal component analysis on the merged gene expression profiles of salivary gland before and after normalization and batch correction. Figure S2. Functional enrichment map for up-regulated DEG. Figure S3. Identification of the optimal number of clusters. Figure S4. Hierarchical clustering of pathway enrichment profiles from patients with SjS and SjS-like mouse models. Figure S5. Correlation between two key pathways enrichment score and KDGs expression values. Figure S6. Enrichment and leading-edge genes of the B cell receptor signaling pathway and B cell activation. Figure S7. Details on the KDGs and the leading edge genes from the B cell receptor signaling pathway and B cell activation. [file 13075_2019_2082_MOESM1_ESM.xlsx]

**Supplementary Figures**


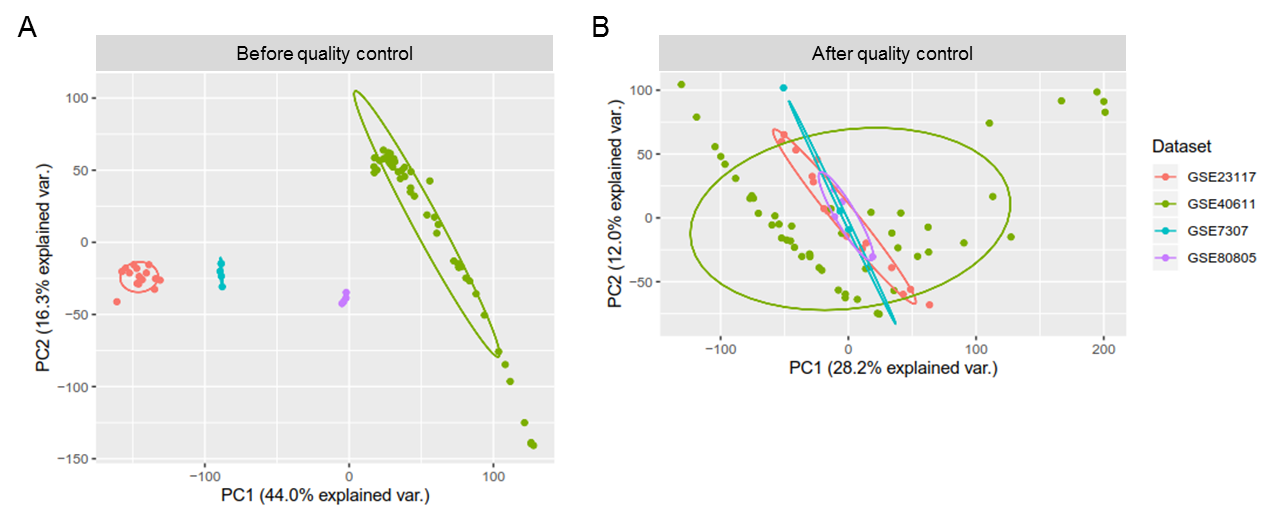


**Figure S1.** Principal component analysis on the merged gene expression profiles of salivary gland before (**A**) and after (**B**) normalization and batch correction.


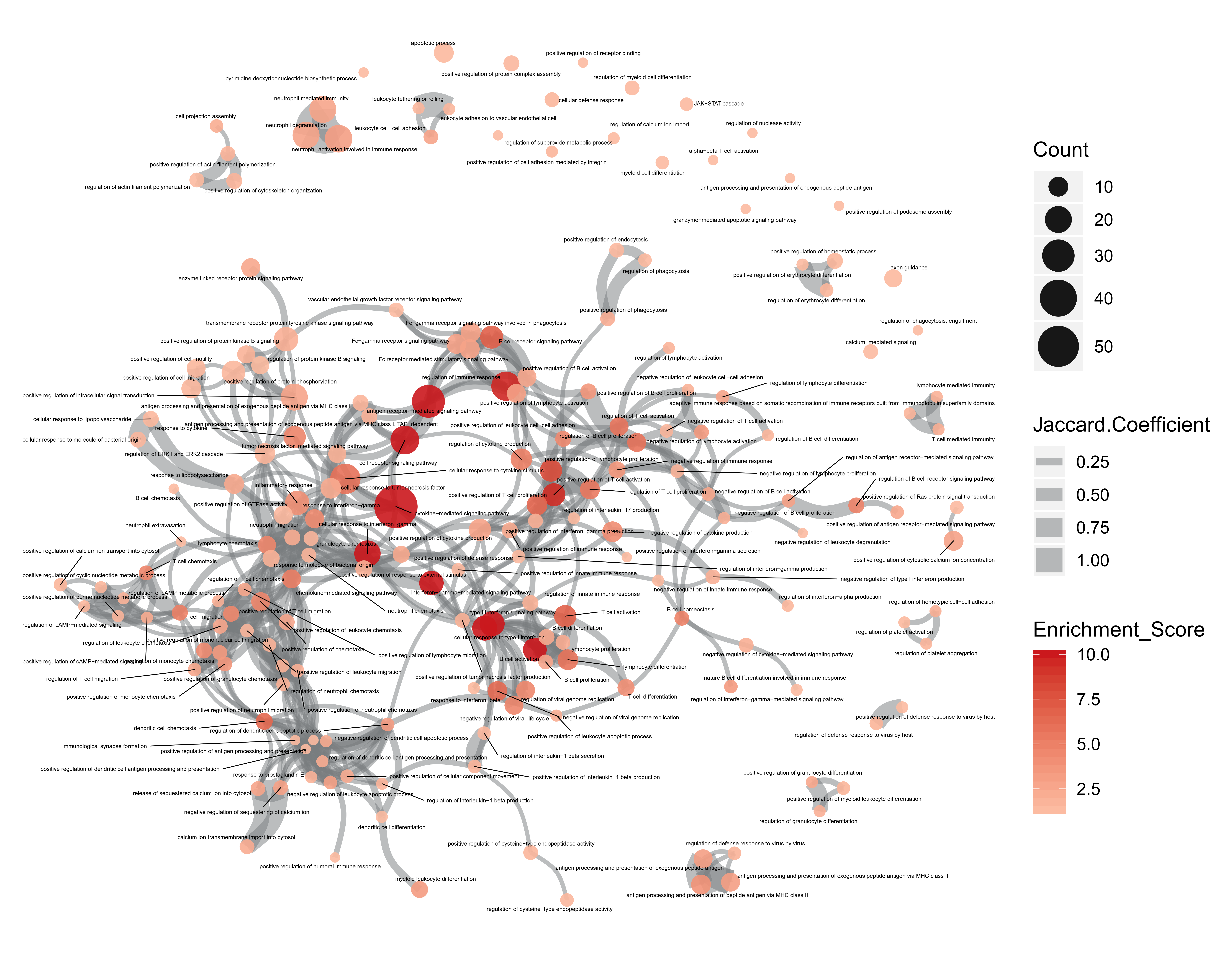


**Figure S2.** Functional enrichment map for up-regulated DEG. Same as **Figure 2B** except for detailed annotation. Magnify the figure to clearly view the labels.


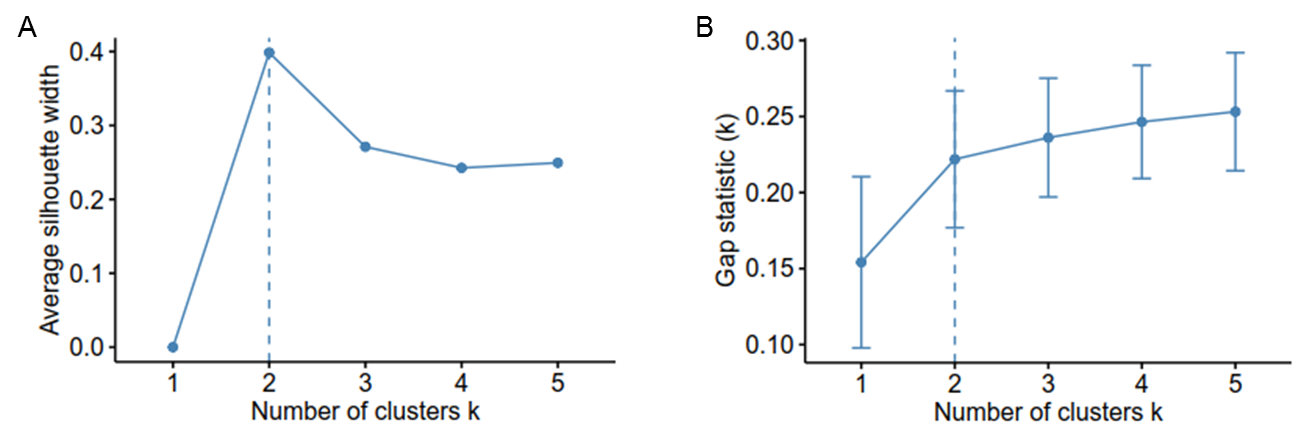


**Figure S3.** **Identification of the optimal number of clusters.** Changes of average silhouette width and gap statistic at rank 2 to 5. Average Silhouette measures the quality of a clustering and determines how well each object lies within its cluster. A high average silhouette width indicates a good clustering. The gap statistic compares the total within intra-cluster variation for different values of *k* with their expected values under null reference distribution of the data. The estimate of *k* is the smallest *k* at which the difference between these ratios at *k* and *k*+1 is greater than its standard deviation.


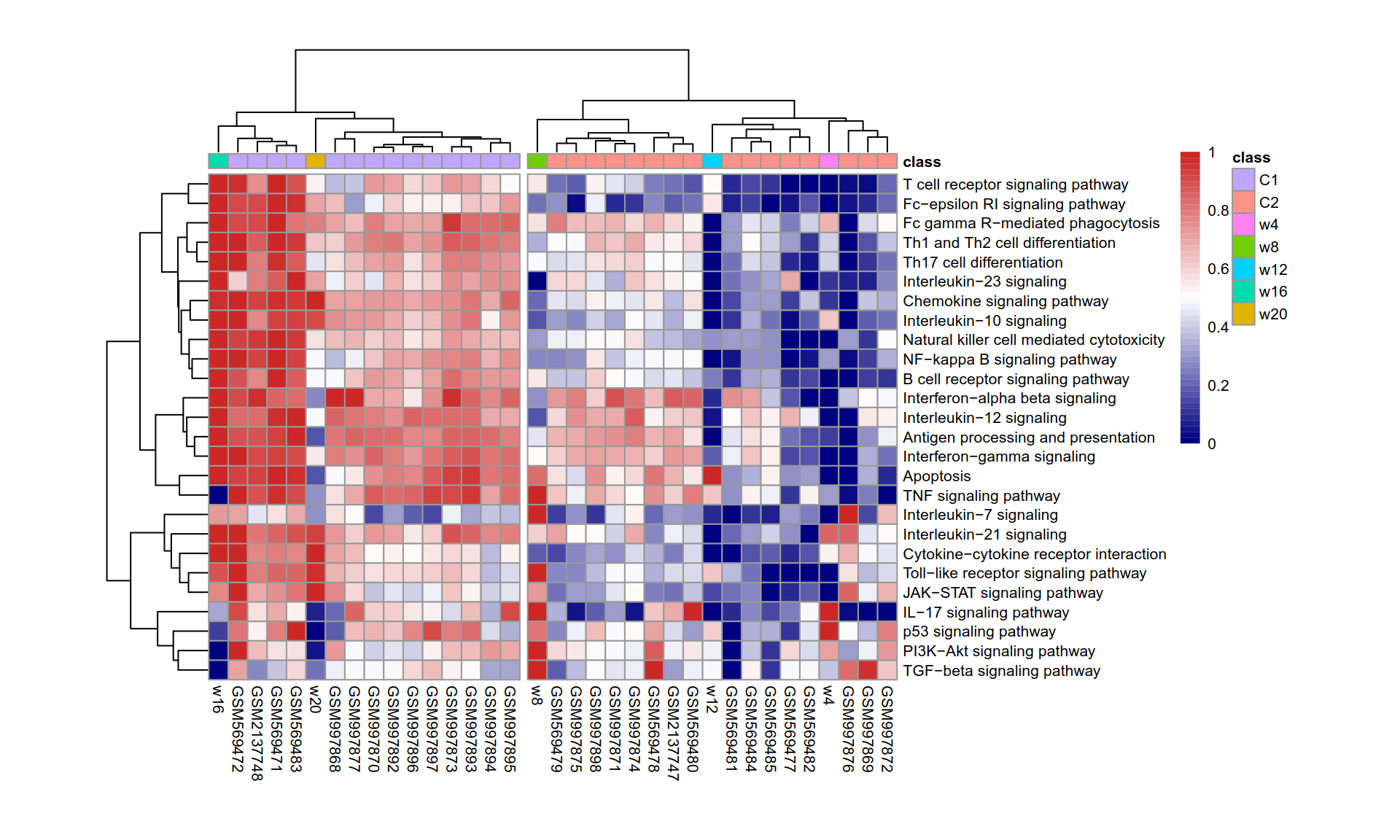


**Figure S4. Hierarchical clustering of pathway enrichment profiles from patients with SjS and SjS-like mouse models.** Data from the SjS-like mouse models (C57BL/6.NOD-*Aec1Aec2* mouse) were equally spaced by 5 time points (4, 8, 12, 16, and 20 weeks). The pathway enrichment profiles of week 4, 8, and 12 were clustered on the cluster 2 (C2), and those of week 16 and 20 were combined with the cluster 1 (C1).


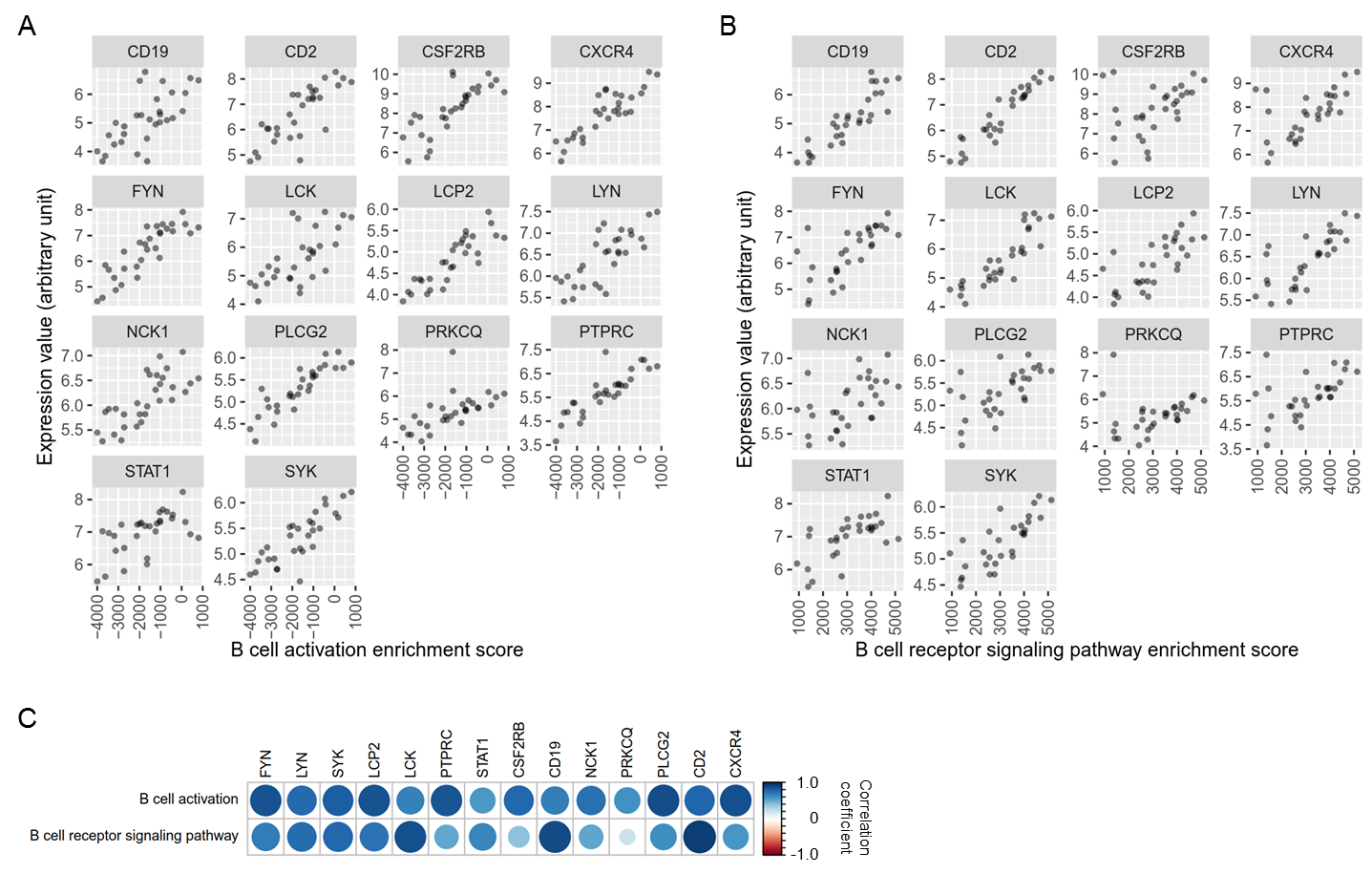


**Figure S5. Correlation between two key pathways enrichment score and KDGs expression values.** Two key pathways from the core SjS module, LCC, were B cell receptor signaling pathway and B cell activation. Correlation analysis was carried out using Pearson’s correlation coefficient. (**A**) Correlation with B cell activation enrichment score. (**B**) Correlation with BCR signaling pathway enrichment score. (**C**) Correlation coefficients of KDGs expression levels with BCR signaling pathways and B cell activation enrichment scores. All but between BCR signaling pathway and PRKCQ were significantly and positively correlated (*P*<0.05).


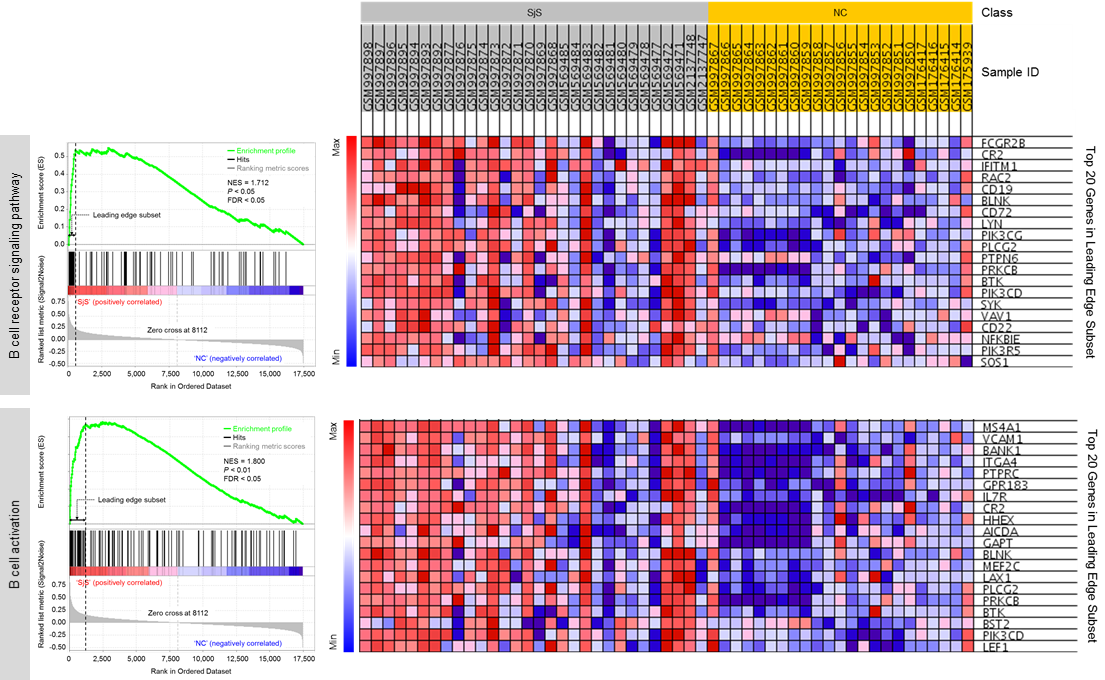


**Figure S6. Enrichment and leading-edge genes of the B cell receptor signaling pathway and B cell activation.** Enrichment plot generated by GSEA analysis of ranked gene expression data (left, up-regulated [red]; right, down-regulated [blue]) by the B cell receptor signaling pathway and B cell activation in SjS versus NC. GSEA-derived heatmap of the top 20 leading-edge genes showing the strongest up-regulation in SjS versus NC based on a signal/noise ratio (SNR) score.


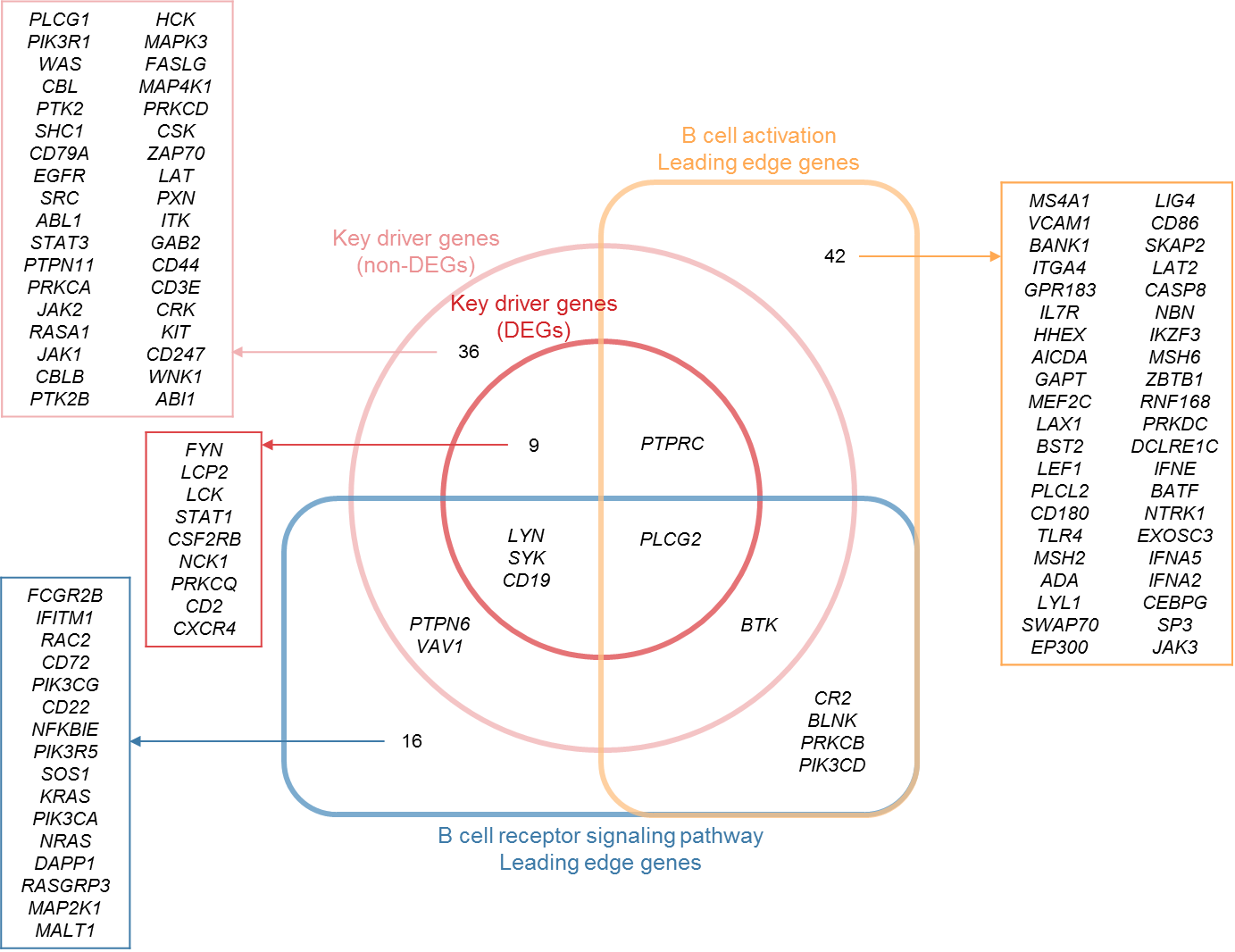


**Figure S7. Details on the KDGs and the leading edge genes from the B cell receptor signaling pathway and B cell activation.** Intersected and distinct subsets of each domain was depicted by Venn Diagram.
