# Supplementary material for: Integrated systems analysis of salivary gland transcriptomics reveals key molecular networks in Sjögren’s syndrome
Source: Arthritis Res Ther. 2019 Dec 19;21:294. doi: 10.1186/s13075-019-2082-9 (PMC6921432; doi:10.1186/s13075-019-2082-9)
Supplement: Supplementary file 2 — Additional file 2. A full list of differentially expressed genes (up-regulated and down-regulated). [file 13075_2019_2082_MOESM2_ESM.pdf]

SUPPLEMENTARY FIGURES

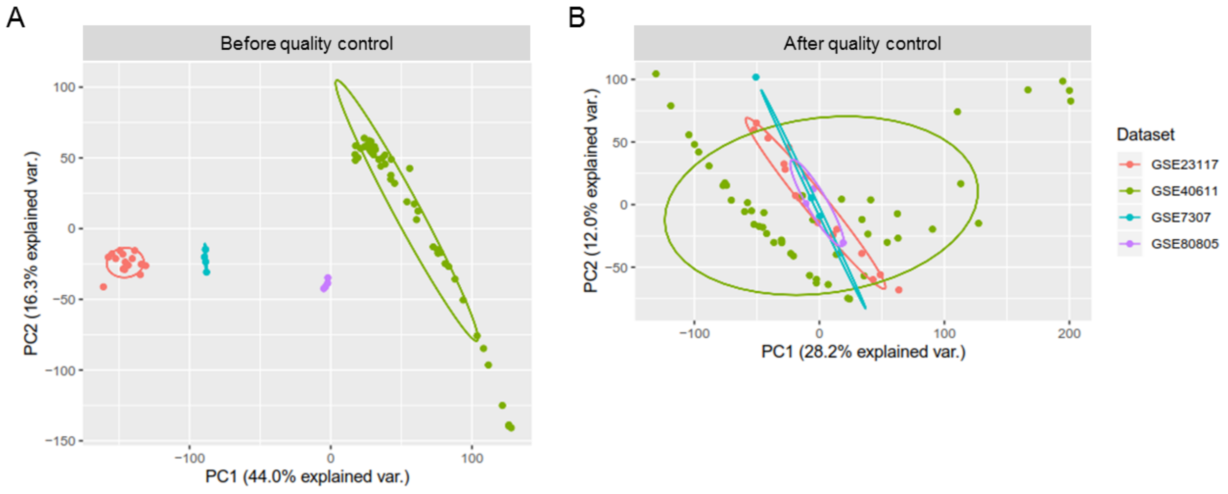

Figure S1. Principal component analysis on the merged gene expression profiles of salivary gland before (**A**) and after (**B**) normalization and batch correction.

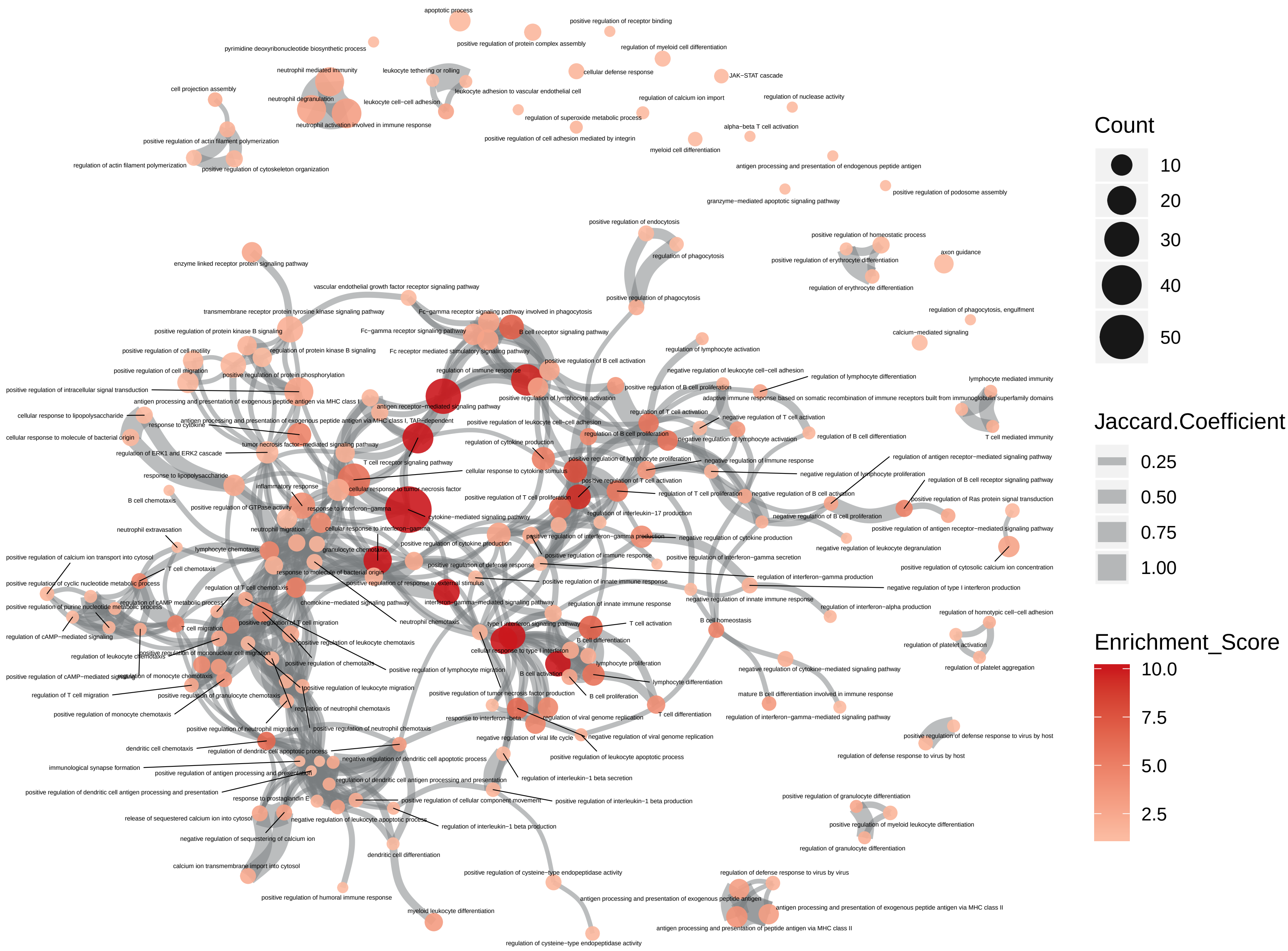

Figure S2. Functional enrichment map for up-regulated DEG. Same as **Figure 2B** except for detailed annotation.

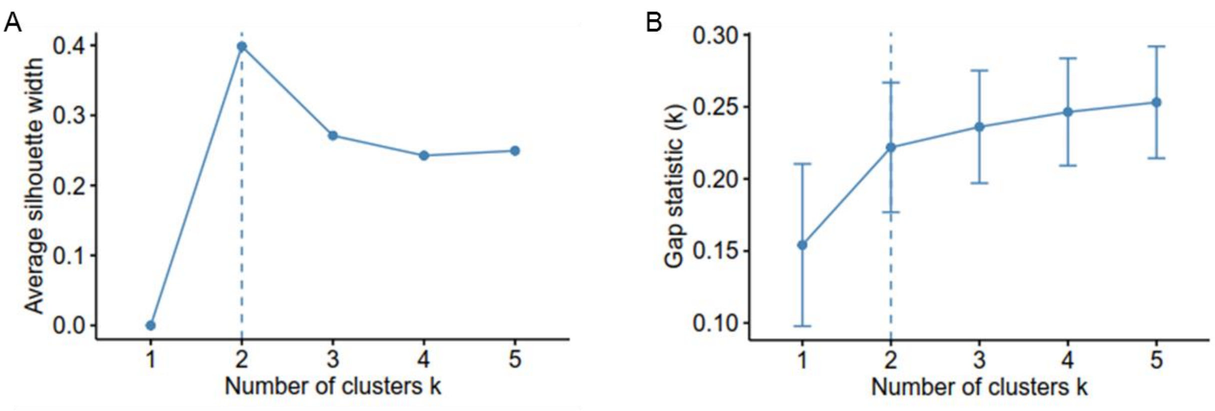

Figure S3. Changes of average silhouette width and gap statistic at rank 2 to 5. Average Silhouette measures the quality of a clustering and determines how well each object lies within its cluster. A high average silhouette width indicates a good clustering. The gap statistic compares the total within intra-cluster variation for different values of k with their expected values under null reference distribution of the data. The estimate of k is the smallest k at which the difference between these ratios at k and k+1 is greater than its standard deviation.

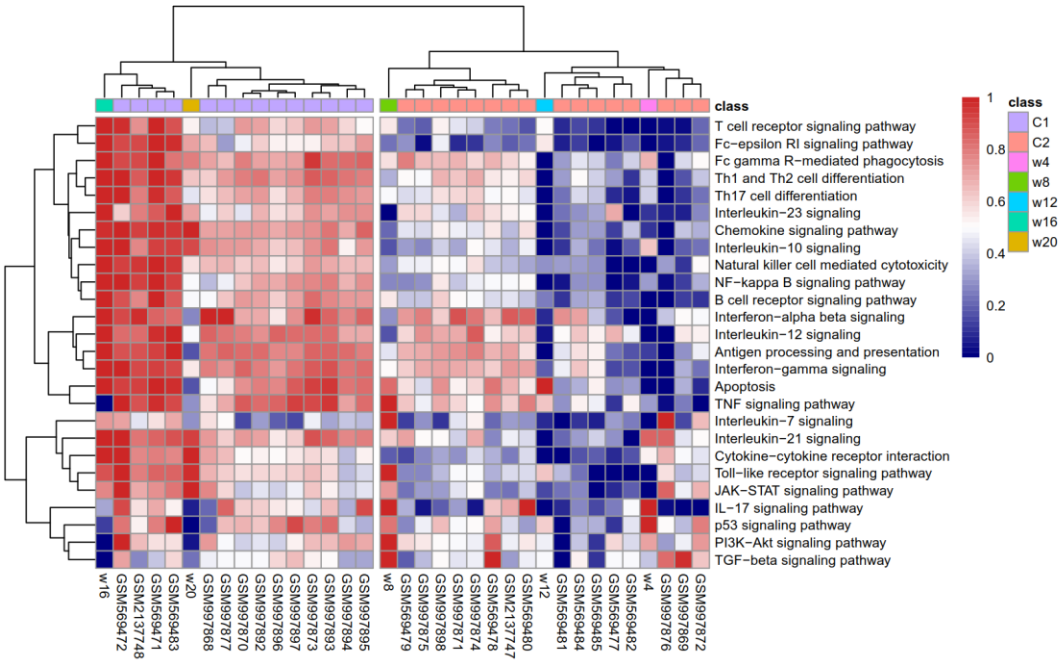

Figure S4. Hierarchical clustering of pathway enrichment profiles from patients with SjS and SjS-like mouse models. Data from the SjS-like mouse models (C57BL/6.NOD-*Aec1Aec2* mouse) were equally spaced by 5 time points (4, 8, 12, 16, and 20 weeks). The pathway enrichment profiles of week 4, 8, and 12 were clustered on the cluster 2 (C2), and those of week 16 and 20 were combined with the cluster 1 (C1).

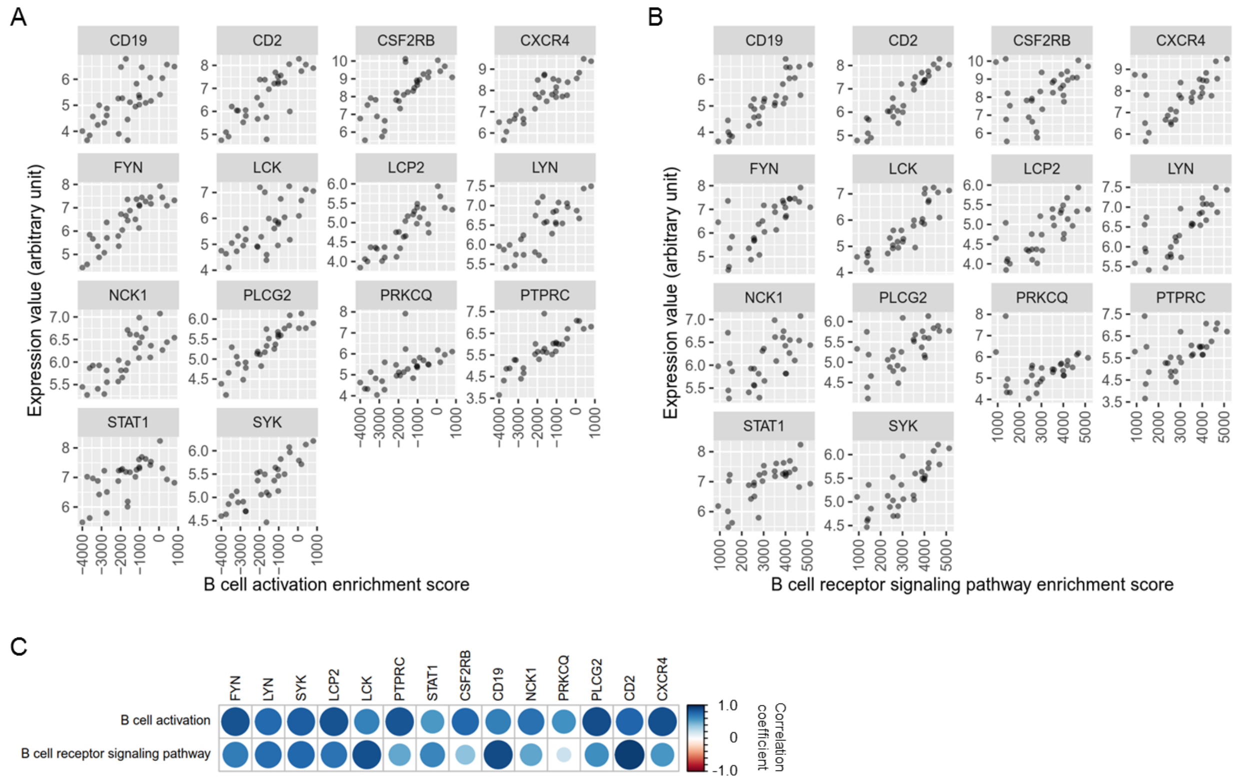

Figure S5. Correlation between two key pathways enrichment score and KDGs expression values. Two key pathways from the core SjS module, LCC, were B cell receptor signaling pathway and B cell activation. Correlation analysis was carried out using Pearson's correlation coefficient. (**A**) Correlation with B cell activation enrichment score. (**B**) Correlation with BCR signaling pathway enrichment score. (**C**) Correlation coefficients of KDGs expression levels with BCR signaling pathways and B cell activation enrichment scores. All but between BCR signaling pathway and PRKCQ were significantly and positively correlated (P<0.05).

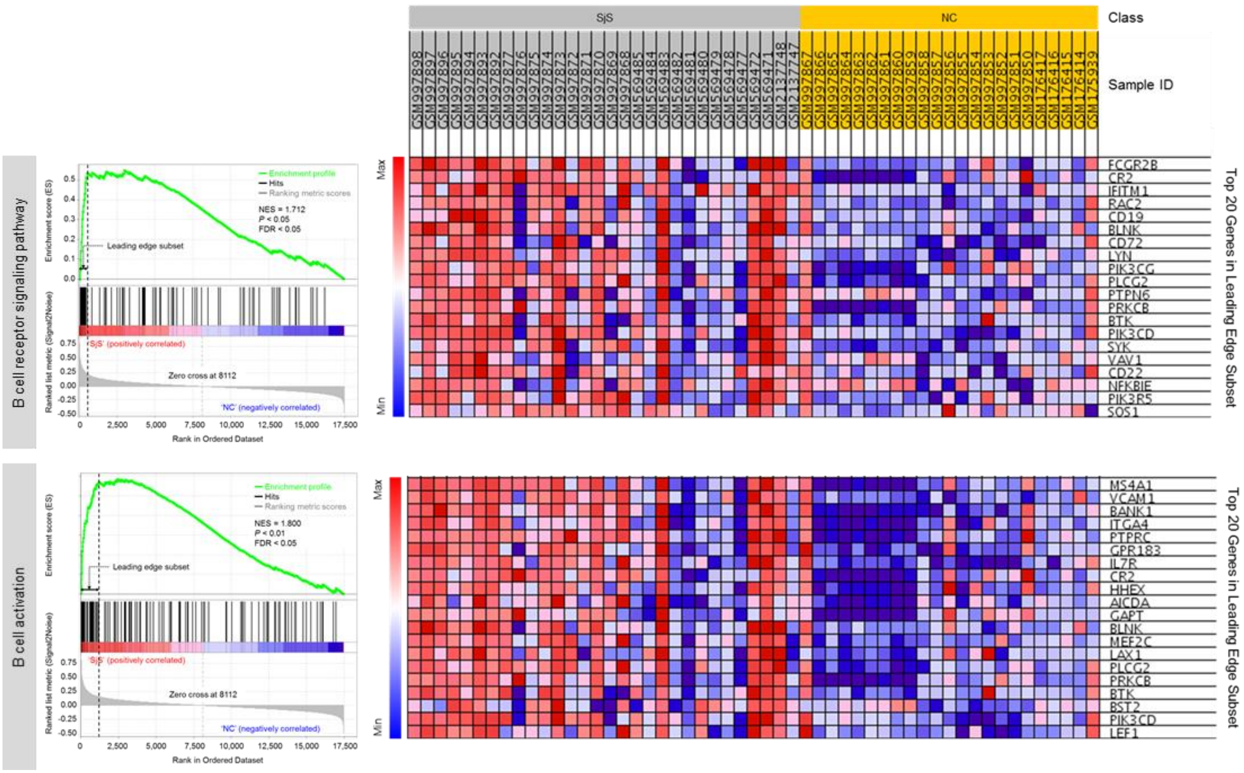

Figure S6. Enrichment and leading-edge genes of the B cell receptor signaling pathway and B cell activation. Enrichment plot generated by GSEA analysis of ranked gene expression data (left, up-regulated [red]; right, down-regulated [blue]) by the B cell receptor signaling pathway and B cell activation in SjS versus NC. GSEA-derived heatmap of the top 20 leading-edge genes showing the strongest up-regulation in SjS versus NC based on a signal/noise ratio (SNR) score.

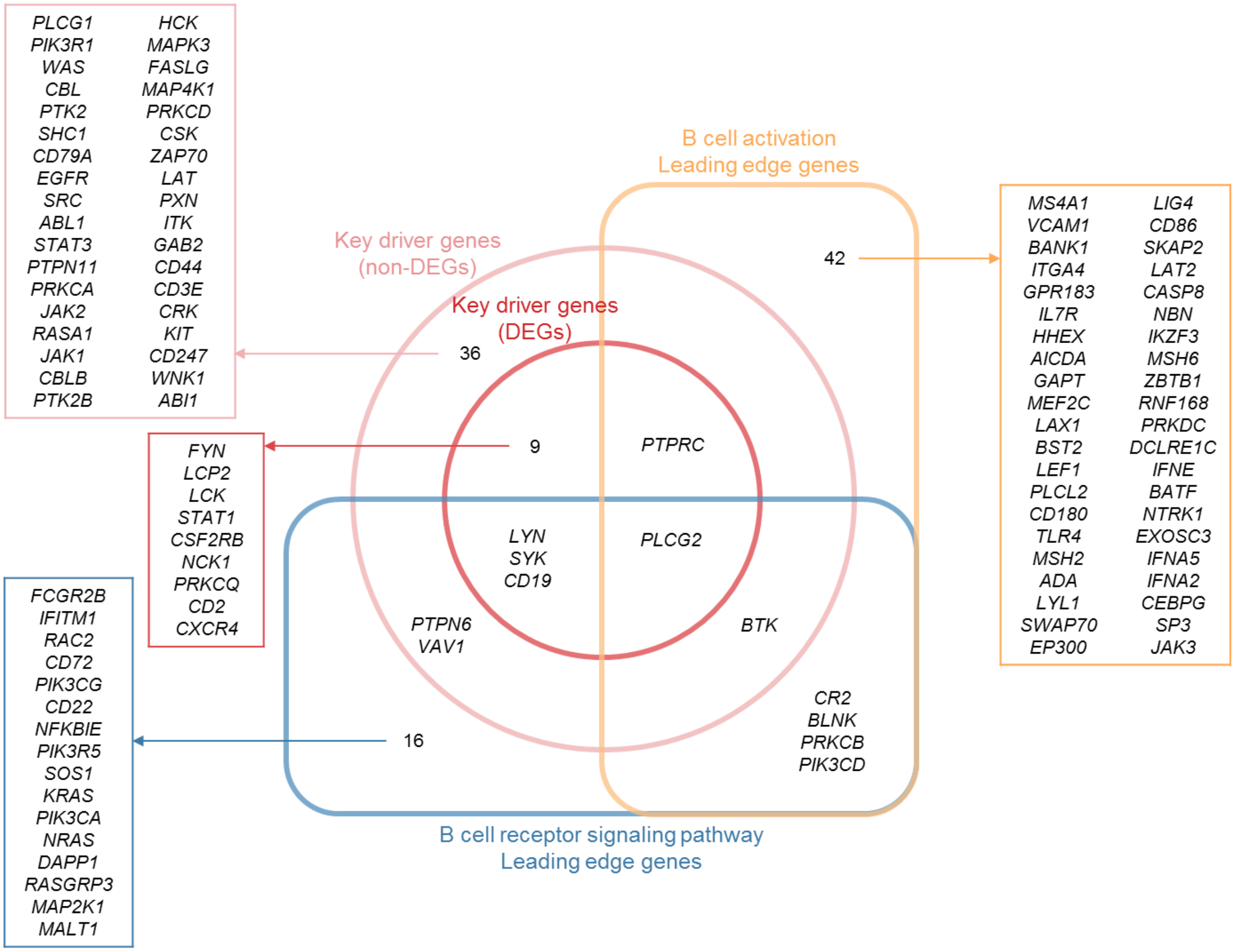

Figure S7. Details on the KDGs and the leading edge genes from the B cell receptor signaling pathway and B cell activation. Intersected and distinct subsets of each domain was depicted by Venn Diagram.
